# Assessing the quality of informed consent in a resource-limited setting: A cross-sectional study

**DOI:** 10.1186/1472-6939-13-21

**Published:** 2012-08-21

**Authors:** Ronald Kiguba, Paul Kutyabami, Stephen Kiwuwa, Elly Katabira, Nelson K Sewankambo

**Affiliations:** 1 Makerere University College of Health Sciences, Kampala, Uganda

## Abstract

**Background:**

The process of obtaining informed consent continues to be a contentious issue in clinical and public health research carried out in resource-limited settings. We sought to evaluate this process among human research participants in randomly selected active research studies approved by the School of Medicine Research and Ethics Committee at the College of Health Sciences, Makerere University.

**Methods:**

Data were collected using semi-structured interviewer-administered questionnaires on clinic days after initial or repeat informed consent procedures for the respective clinical studies had been administered to each study participant.

**Results:**

Of the 600 participants interviewed, two thirds (64.2%, 385/600) were female. Overall mean age of study participants was 37.6 (SD = 7.7) years. Amongst all participants, less than a tenth (5.9%, 35/598) reported that they were not given enough information before making a decision to participate. A similar proportion (5.7%, 34/597) reported that they had not signed a consent form prior to making a decision to participate in the study. A third (33.7%, 201/596) of the participants were not aware that they could, at any time, voluntarily withdraw participation from these studies. Participants in clinical trials were 50% less likely than those in observational studies [clinical trial vs. observational; (odds ratio, OR = 0.5; 95% CI: 0.35-0.78)] to perceive that refusal to participate in the parent research project would affect their regular medical care.

**Conclusions:**

Most of the participants signed informed consent forms and a vast majority felt that they received enough information before deciding to participate. On the contrary, several were not aware that they could voluntarily withdraw their participation. Participants in observational studies were more likely than those in clinical trials to perceive that refusal to participate in the parent study would affect their regular medical care.

## Background

With the growing volume of clinical and public health research in developing countries, increasing attention is now focused on how existing research regulatory mechanisms are performing in protecting study participants [1]. In these countries, study participants’ socioeconomic status, education level as well as health conditions have been found to render them vulnerable [2-4] to exploitation [5].

To protect such participants, it necessitates establishment of functioning independent ethics review committees to evaluate the research protocols before recruitment commences [6]. These committees present the first and a principal step for independent review of the scientific and ethical quality of research proposals before their implementation. That notwithstanding, informed consent continues to be a contentious issue in clinical and public health research carried out in resource-limited settings [7].

The assumption that the approved research protocol is predictive of the actual implementation process may not necessarily be true thus requiring ethics committees to exercise their responsibility of monitoring the conduct of ongoing research projects and the consent process [8]. However, human and non-human resource constraints coupled with an increase in the volume of research projects conducted may present logistical challenges in the execution of this function [7,9-12]. Whereas available guidelines prescribe specific ethical standards for the informed consent process [13-17] circumstances may arise in which these standards are not explicit [7] resulting in variable interpretation of this process at the time of research project implementation.

Without routine monitoring of research activities, the ambiguity of ethical standards in the consenting process may create room for compromise and laxity among research project implementers resulting in violation of the rights and welfare of research participants [18].

To our knowledge, a few studies have explored the quality of informed consent in resource-limited settings [3,19-23]. However, there is a paucity of published literature on this subject in the Ugandan setting. This study aimed to evaluate the informed consent process among human research participants in randomly selected active research projects approved by the School of Medicine Research and Ethics Committee at the College of Health Sciences, Makerere University, Kampala, Uganda.

## Methods

We conducted a cross-sectional study over a period of one month from 28^th^ July 2008 to 22^nd^ August 2008. Ethical approval for this study was granted by the School of Medicine Research and Ethics Committee (SOMREC), Makerere University College of Health Sciences.

### Study sample

Study participants involved in the consent evaluation process were drawn from clinical trials and observational studies that had received ethical clearance from the SOMREC. All selected studies were conducted at the Mulago National Referral Hospital, and the Infectious Diseases Institute, both of which are located adjacent to the Makerere University College of Health Sciences. Eight clinical trials and seven observational studies were selected. All selected clinical trials were treatment trials among HIV-infected patients. Treatments tested included antiretroviral therapy, Herpes Simplex Virus type 2 (HSV-2) suppression, antibiotics, antimalarials, and computerized cognitive rehabilitation training. All selected observational studies (except one specific to Tuberculosis) were also focused on HIV-infected patients. These included observations of renal features in HIV-infected patients, studies of CD4 trends, immune reconstitution inflammatory syndrome after commencing antiretroviral therapy, and HIV-1 co-receptor tropisms.

The format of informed consent forms for all selected studies adhered to the guidelines recommended by the SOMREC which, at the minimum, stipulate inclusion of sections on purpose of the research, study procedures, discomforts and risks, potential benefits, privacy and confidentiality, compensation for participation, voluntary participation, investigators’ contact information for questions about study, and ethics committee contact for questions about rights and welfare of participants. Readability levels of the English versions of consent forms were set at primary school level (grade level 6) and translated into the mainly spoken local language “*Luganda*” for participants who preferred the local language and those who did not have any formal education. Informed consent was obtained from each participant prior to recruitment into our study. The required sample size of 600 participants was obtained by consecutively sampling study participants from each of the selected active clinical studies.

### Data collection methods

Data were collected by five research assistants on research clinic days after initial or repeat informed consent procedures for the respective clinical studies had been administered to each study participant. We used semi-structured interviewer-administered questionnaires to collect the data. The research assistants were supervised throughout the process of data collection.

### Data management and analysis

Quantitative data were entered into EpiData version 3.1 and exported to SPSS 12.0 for statistical analysis. The results are presented as simple proportions, means, frequencies, bar charts, and odds ratios with their 95% confidence intervals. The level of significance was set at P ≤ 0.05.

## Results

### Study population

**Figure 1:**
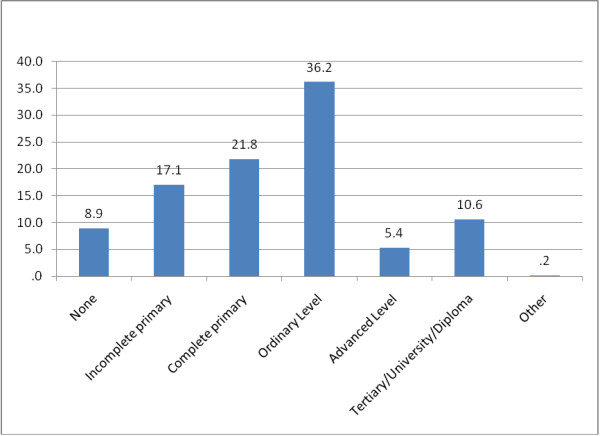
Highest Level of Education attained among 597 participants.

The overall mean age of respondents was 37.6 (SD = 7.7) years and about two-thirds (64.2%, 385/600) of them were female (Table 1). The mean age of respondents enrolled in clinical trials (38.2, SD = 7.5) was statistically significantly higher than that of respondents in observational studies (36.7, SD = 7.3) (Table 2). Whereas just under a tenth (8.9%, 53/597) of individuals had never received any formal education, more than a half (52.4%, 313/597) had, at least, attained secondary level education (Figure 1). However, respondents in observational studies were almost twice as likely to have no formal education as compared to those in clinical trials and this difference was significant (P = 0.001) (Table 2). Ten per cent (10%, 60/598) of participants were initial consenters while the rest (90%, 538/598) were repeat consenters. In this survey, 5.9% (35/598) of participants reported that they were not given enough information prior to making a decision to participate in the study.

**Table 1:** Characteristics of study participants

	
**Sociodemographic characteristics**	
Age, mean (SD), yr	37.6 (7.7)
No formal education (n, %)	53 (8.9)
Female Gender (n, %)	385 (64.2)
**Informed Consent Process**	**No**
First research project for participant	8.0 % (48/600)
Felt were given enough information*	5.9 % (35/598)
Signed a consent form*	5.7 % (34/592)
Felt pressured to participate*	95.0 % (568/598)
Refusal to participate would affect regular medical care*	59.8 % (357/597)
Anticipated benefits for participation*	7.0 % (42/599)
Anticipated risks for participation	68.2 % (409/600)
Knowledge of voluntary withdrawal*	33.7 % (201/596)

*The numbers do not add up to 600 participants due to missing data.

**Table 2:** Characteristics of study participants stratified by clinical trial vs. observational study

**Sociodemographic characteristics**			
	**Clinical Trial**^**±**^	**Observational**^**±**^	**P-value**
**Mean age (mean, SD), yr**	**38.2 (7.5)**	**36.7 (7.3)**	**0.027**
**No formal education (n, %)**	**39 (16.6)**	**81 (30.7)**	**0.001**
Female Gender (n, %)	146 (62.1)	173 (65.8)	NS
**Informed Consent Process**	**No**		**aOR**
First research project for participant*	5.1 % (12/235)	8.3 % (22/264)	NS
Felt were given enough information*	**3.0 % (7/235)**	**7.3 % (19/262)**	**0.3(0.13-0.84)**
Signed a consent form*	4.3 % (10/234)	6.6 % (17/258)	NS
Felt pressured to participate*	99.1 % (232/234)	95.1 % (251/264)	NS
Refusal to participate would affect regular medical care*	**48.5 % (113/233)**	**65.4 % (172/263)**	**0.5(0.35-0.78)**
Anticipated benefits for participation*	1.7 % (4/235)	2.7 % (7/263)	NS
Anticipated risks for participation*	68.9 % (162/235)	65.9 % (174/264)	NS
Knowledge of voluntary withdrawal*	28.8 % (67/235)	36.4 % (96/264)	NS

*The overall numbers in rows do not add up to 600 participants due to missing data.

^**±**^ Data on clinical trial vs. observational study status were available for 499 out of 600 participants with 235 in clinical trials and 264 in observational studies.

aOR Odds Ratio adjusted for age, gender, educational level and whether participant was involved in an initial or repeat consent process.

On a visual analogue scale (0 to 10 arbitrary scores, 0 = not satisfied at all, 10 = fully satisfied), close to half (45.4%, 271/597) recorded a score of 10 indicating that they were fully satisfied with the informed consent process. The mean satisfaction score was 8.73 (SD = 1.57) and the data were normally distributed. There was no statistically significant difference between mean scores of initial versus repeat consenters. Amongst the reasons reported for not signing consent forms, participants (73.5%, 25/34) reported that they were not given a form to sign (72%, 18/25), received the form but did not want to sign (20%, 5/25), and did not know the reason (8%, 2/25). Of 598 (99.7%) out of 600 respondents, 568 (95%) indicated that they felt no pressure to participate in the parent study, 28 (4.7%) felt pressured to participate, while 2 (0.3%) were not sure. Amongst individuals who signed a consent form, a comparable proportion (4.5%) of subjects felt pressured to participate in the parent study. Whereas none of the individuals who did not sign a consent form reported that they felt pressured to enroll in study, 5.9% (2/34) were not sure if they were pressured or not.

Overall, the majority (93%, 557/599) of respondents believed that there were personal benefits for participating in the study. Thirty (5%) individuals were not sure whether they would receive benefits for participation in the study while 12 (2%) did not expect any benefits. Ninety six per cent (96.2%, 226/235) of participants in clinical trials and 91.3% (240/263) of those in observational studies expected benefits but these proportions were not significantly different (P = 0.07). Amongst those who expected benefits, the most frequently cited benefits were direct (81.7%) while the others cited were indirect (18%). Direct benefits mentioned related to medical gains derived from administered interventions while indirect benefits related to greater access to professional health care. A little more than two-thirds (68.2%, 409/600) of respondents believed that there were no risks for participation in the study. The most frequently reported risks were physical (60.2%) followed by psychological (23.1%), economic (11.1%), and social harm (5.6%). A third (33.7%, 201/596) of the participants were not aware that they could, at any time, voluntarily withdraw participation from these studies.

Respondents in clinical trials were 50% less likely to perceive that refusal to participate in the study would affect their regular medical care [clinical trial vs. observational; (OR = 0.5; 95% CI: 0.35-0.78)] (Table 2).

## Discussion

This research study elicited information on the adequacy of the application of the basic elements in obtaining informed consent in a range of research studies conducted among participants in our setting. The majority of participants reported that they were given enough information to participate in the research studies and similar results have been obtained elsewhere [2]. This finding was corroborated with the very high overall informed consent process satisfaction scores. In addition, the largest proportion of participants did not feel pressured to participate in the research studies.

Sanchez *et al.*[20] observed that before taking part in a clinical trial, participants assessed perceived benefits and risks, their rights and responsibilities as well as their understanding of what research and volunteering meant. Processing this information before deciding to enroll enhanced commitment to the study and promoted participants’ right to choose without coercion [20]. One study in the developed world however, indicated that the information participants received did not have to be ‘very good’ or even ‘good’ to be perceived as adequate [24]. Moreover many of those who reported that they had received adequate information did not know or even remember about the liberty to withdraw [24]. Conversely, participants in developing country settings may fail to appropriately process this information thus affecting free choice due to low awareness of their rights, their scarce questioning of risks involved [20], power relations between them and the researcher [19], and failure to understand that participation has a research purpose apart from whatever benefits that might arise [2,25].

Participants in observational studies, however, were more likely to report that they were not given enough information before making a decision to participate when compared to those in clinical trials. This may be interpreted to suggest that investigators probably go an extra mile to provide information during the rigorous process of recruiting participants for clinical trials. Investigators are compelled to do more due to the stringent regulatory framework, the complex nature, and risk attributed to clinical trials. This may also partly explain why participants in clinical trials were less likely than those in observational studies to perceive that refusal to participate in the study would affect their regular medical care.

Overall, almost all (including all initial consent) participants believed that there were benefits for participating in the clinical study but only about one third believed that there were risks. This observation, which may suggest therapeutic misconception, has been described elsewhere [26,27] and is consistent with other studies in a developing country setting where participants anticipated improved clinical care and downplayed the risks associated with clinical research [28].

Amongst those who expected benefits, the most frequently cited benefits were direct while a few cited were indirect. Direct benefits mentioned related to medical gains derived from administered interventions while indirect benefits related to greater access to professional health care. In randomised clinical trials, it is important to explain to research participants that they have an equal chance of being assigned to either the intervention arm or to the comparison arm and that at the onset of a well designed randomized clinical trial, it is not known which arm will provide direct benefit. However, whether or not participants receive the experimental intervention, they may receive indirect benefits in the form of more attention from health worker, closer follow-up, and screening tests or additional tests to determine outcomes. These collateral benefits might lead to improved clinical outcomes even in situations where the intervention is no better than standard of care[29,30]. In some observational study designs, especially surveys and reviews of existing medical records, there are no direct benefits but research participants may receive indirect benefits as is the case with clinical trials.

It is worth noting that up to a third of participants were not aware that they could voluntarily withdraw their participation at any time despite the fact that all the studies selected had incorporated this section in their consent forms. Conversely, a study on comprehension of informed consent in rural and peri-urban Mali observed that 90% of respondents did not understand withdrawal criterion [25]. This finding is not limited to developing countries since similar experiences have been reported in the developed world [24]. Lynoe *et al.* observed that deficiencies in participants' perception of information during the consent process may be caused by informers rather than the participants [24]. This may partly explain why participants in clinical trials who are usually taken through more rigorous consent processes by informers are less likely than those in observational studies to perceive that refusal to participate may affect their regular medical care. However, participants in observational studies were almost twice as likely to have no formal education as compared to those in clinical trials and this may also explain their higher likelihood to perceive that they would be denied regular medical care if they turned down an invitation to take part in the research studies. This finding is consistent with results obtained elsewhere which observed that level of education is a key factor in determining the outcome of the informed consent process[3,4].

One in every 20 participants did not sign a consent form. The majority of them reported that they were not given the consent form to sign. This is a violation of Good Clinical Practice (GCP) standards which stipulate that, whenever consent is not waived, every **research** participant or **the research participant’s representative** must sign and date the consent document [31]. If **the** participant **i**s **illiterate,** a thumbprint should be used instead [31]**.** This underlines the need for ethics committees to conduct study site visits as they execute their role of research oversight, although more capacity building will be required for ethics committees in resource-limited settings [10-12] to fully exercise this mandate.

Relevant empirical data is required to inform the overall policy efforts focused towards protecting the rights and welfare of study participants in resource-limited settings and our study aimed to contribute towards this cause.

This study has several important limitations. First, we did not investigate in-depth the recall and comprehension aspects of informed consent in the selected clinical studies. This would have facilitated a better assessment of risk factors for poor consenting processes in our setting. Second, we used self-report for all our measurements which may have yielded socially desirable answers from participants. Third, the median duration of participants in the research studies was 2 years. Such participants may have undergone several sessions of informed consent and may not be representative of the responses that would have been obtained from first-time participants recently enrolled in clinical studies. Stratified analyses attempted to identify differences between initial and repeat consent participants and the two groups appeared to differ only if participants were enrolled into their first ever parent study.

## Conclusions

Most of the participants signed informed consent forms and the majority felt that they had received enough information before deciding to participate in the parent studies. On the contrary, not all were aware that they could voluntarily withdraw their participation. Participants in observational studies were more likely than those in clinical trials to perceive that refusal to participate in the parent study would affect their regular medical care.

## Competing interests

The authors declare that they have no competing interests.

## Authors’ contributions

RK participated in the conception, design and coordination of the study as well as performed the statistical analysis and drafted the manuscript. PK and SK conceived of the study and participated in its design in conjunction with EK and NKS. All authors read and approved the final manuscript.

## Pre-publication history

The pre-publication history for this paper can be accessed here:

http://www.biomedcentral.com/1472-6939/13/21/prepub
